# The Effects of Dietary Glycine on the Acetic Acid-Induced Mouse Model of Colitis

**DOI:** 10.1155/2020/5867627

**Published:** 2020-08-05

**Authors:** Xin Wu, Yongmin Zheng, Jie Ma, Jie Yin, Shuai Chen

**Affiliations:** ^1^College of Animal Science and Technology, Hunan Agriculture University, Changsha, China; Hunan Co-Innovation Center of Animal Production Safety, Changsha, Hunan 410128, China; ^2^Scientific Observing and Experimental Station of Animal Nutrition and Feed Science in South-Central, Ministry of Agriculture, Hunan Provincial Engineering Research Center of Healthy Livestock, Key Laboratory of Agro-Ecological Processes in Subtropical Region, Institute of Subtropical Agriculture, Chinese Academy of Sciences, Changsha, Hunan 410125, China

## Abstract

Inflammatory bowel disease, a gut disease that is prevalent worldwide, is characterized by chronic intestinal inflammation, such as colitis, and disorder of the gut microbiome. Glycine (Gly) is the simplest amino acid and functions as an anti-inflammatory immune-nutrient and intestinal microbiota regulator. This study aimed at investigating the effect of Gly on colitis induced in mice by intrarectal administration of 5% acetic acid (AA). Bodyweight and survival rates were monitored, and colonic length and weight, serum amino acid concentrations, intestinal inflammation-related gene expression, and colonic microbiota abundances were analyzed. The results showed that Gly dietary supplementation had no effect on the survival rate or the ratio of colonic length to weight. However, Gly supplementation reversed the AA-induced increase in serum concentrations of amino acids such as glutamate, leucine, isoleucine, and valine. Furthermore, Gly inhibited colonic gene expression of interleukin- (IL-) 1*β* and promoted IL-10 expression in colitis mice. Gly supplementation also reversed the AA-induced reduction in the abundance of bacteria such as Clostridia, Ruminococcaceae, and Clostridiales. This change in the intestinal microbiota was possibly attributable to the changes in colonic IL-10 expression and serum concentrations of valine and leucine. In sum, Gly supplementation regulated the serum concentrations of amino acids, the levels of colonic immune-associated gene expression, and the intestinal microbiota in a mouse model of colitis. These findings enhance our understanding of the role of Gly in regulating metabolism, intestinal immunity, and the gut microbiota in animals afflicted with colitis.

## 1. Introduction

Inflammatory bowel disease (IBD), including Crohn's disease (CD) and ulcerative colitis (UC), is characterized by a loss of intestinal mucosal homeostasis associated with inappropriate and aggravated immune responses to intestinal lumen antigens [1–3]. The clinical symptoms of IBD include diarrhea, abdominal pain, bloody stools, tenderness, and abdominal mass. The increased incidence of IBD is a worldwide healthcare problem [4]. It is widely believed that IBD is caused by complex interactions between genetic and environmental factors, such as immune-response disorders and microbial community changes. However, the cause of IBD remains unclear, and further research is needed [5, 6].

Several types of pharmacologically induced animal colitis models have been developed, such as dextran sulfate- (DSS-), trinitrobenzene sulfonic acid- (TNBS-), or acetic acid- (AA-) induced models [7, 8]. DSS is widely used for this purpose, as is TNBS, typically in combination with ethanol. AA causes UC (hereafter termed “colitis”) by damaging the intestinal mucosa epithelium and is a low-cost and convenient technique. Previous studies demonstrated that AA-induced colitis might be a good model with which to study the efficacy of drugs [9], so we used AA to induce colitis in this study.

Anti-inflammatory drugs such as cytokine antagonists and immunosuppressive drugs such as corticosteroids are currently used for IBD treatment [10–12]. However, long-term use of cytokine antagonists may have severe side effects [13]. Therefore, new adjuvant therapies are required to overcome the limitations of current drug therapies [14].

Many amino acids have been shown to have beneficial effects in IBD. For example, arginine reduces mucosal permeability, inhibits inflammation, and increases inducible nitric oxide synthase (iNOS) activity in mice with DSS-induced colitis [15]. It has also been shown that Gly may inhibit cytokine secretion in monocytes, macrophages, and neutrophils, and regulate T cell activities [16]. Dietary Gly prevents diarrhea, bodyweight loss, and ulceration in rats with DSS-induced IBD [17, 18]. However, the effects of Gly on AA-induced IBD in mice remain unclear.

This study aimed at investigating the effect of Gly on AA-induced colitis in mice. The results add to our knowledge of a possible role for Gly in colitis treatment.

## 2. Materials and Methods

### 2.1. Animal Model

This study was conducted following the guidelines of the Animal Welfare Committee of the Institute of Subtropical Agriculture, Chinese Academy of Sciences (2015-8A). Female six-week-old ICR (Institute for Cancer Research) mice were purchased from the SLAC laboratory animal center (Changsha, China). The mice were housed in a temperature-controlled environment with a 12 h light/dark cycle and had free access to food and water. Sixty mice were randomly divided into three groups of 20 animals: a control (Con) group (fed the basal diet), a model (Mod) group (fed the basal diet), and a model+Gly (ModGly) group (fed the basal diet supplemented with 0.1% Gly). The dose of Gly was selected following preliminary experiments. The Con group and Mod group shared the data with our previous study [19]. On the seventh day of the experiment, all of the mice were fasted for 36 h and were intrarectally treated with 5% AA (Mod and ModGly group) or saline solution (Con group). The surviving mice were monitored for 1 week after the administration of AA, and all of the mice were then sacrificed by cervical dislocation, and blood, colons, and colonic contents were collected.

### 2.2. Analysis of Serum Amino Acids

High-performance liquid chromatography (HPLC) was used to analyze serum amino acids. Briefly, 0.1 ml of the serum sample was thoroughly mixed with 4.9 ml of 0.01-M hydrochloric acid and then centrifuged at 5000 rpm and 4°C for 5 min. The supernatant (500 ml) was then removed and incubated at 4°C for 12 h and then homogenized with 8% salicylic acid. The resulting homogenate was centrifuged twice at 12,000 rpm and 4°C for 10 min. Authentic standards (Sigma Chemicals) for quantifying amino acids in serum samples were prepared in 0.01-M hydrochloric acid and stored at -70°C.

### 2.3. RT-PCR

Total RNA was extracted from colonic tissues using the TRIZOL reagent kit (Invitrogen, USA). The PrimeScript RT kit with gDNA Eraser (Takara Bio Inc., Qingdao, China) was used to synthesize cDNA, according to the product manual. Primers (Table 1) used in this study were designed under the principles of primer design using Primer 6.0 software (PRIMER-E, New Zealand) and Oligo 5.0 software (Molecular Biology Insights, Inc., USA), based on the gene sequences of the house mouse (*Mus musculus*) on GenBank. The SYBR Premix Ex Taq kit (Takara, Japan) was used for RT-PCR analysis, and triplicate reactions were performed using the Applied Biosystems 7900HT Fast Real-Time PCR System (Thermo, USA) by the following methods: (1) initial denaturation (90°C 30 s); (2) amplification and quantification (40 cycles at 95°C 5 s, 1 cycle at 60°C 30 s). Relative gene expression was calculated with the formula 2^-(*∆∆*Ct)^.

### 2.4. Gut Microbiota Analysis

16S rDNA sequencing was used for intestinal microbiota analysis, according to our previous study [20]. Digested matter in the colon cavity was collected, and the DNA was extracted from this material using a Qiagen QIAamp DNA Stool Mini kit. Illumina MiSeq sequencing was used to analyse the V3 to V4 regions of bacterial 16S rDNA, and the data analysis was performed by Anoru Genomics Technology Co., Ltd., (Beijing, China). Briefly, the library was generated and sequenced to produce 400 base pair/600 base pair single-end reads. Single-end reads were assigned to the samples based on their unique barcode and truncated by the removal of their barcode and the primer sequence. Sequence analysis and operational taxonomic unit (OTU) clustering were performed using UPARSE software (v7.0.1001, https://drive5.com/usearch/) following quality filtering, and sequences with more than 97% similarity were clustered to the same OTU. RDP Classifier (V2.2, Michigan State University Board of Trustees, East Lansing, MI) was used for species annotations, based on the GreenGene database. MUSCLE software (Version 3.8.31) was used to study the phylogenetic relationships of different OTUs, to examine differences in the dominant species in different samples (groups), and for multiple-sequence alignments. The abundant information in the OTUs was normalized for suvbsequent analysis of the alpha diversity, beta diversity, and the environmental-factor correlation analysis.

### 2.5. Statistical Analyses

GraphPad Prism 6.0 (GraphPad Software, Inc., La Jolla, CA) was used for statistical analyses and to create graphs. Statistical analyses between the Con and Mod groups and the Mod and ModGly groups were performed by *t*-test. Results are shown as mean ± SD. *P* < 0.05 was considered significant; ^∗^*P* < 0.05, ^∗∗^*P* < 0.01, ^∗∗∗^*P* < 0.001.

## 3. Results

### 3.1. Survival Proportions and Ratio of Colon Weight to Length

**Figure 1:**
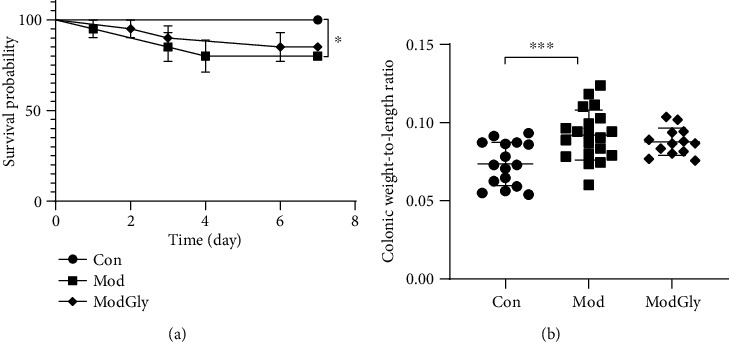
Survival proportions, the ratio of colon weight to length. (a) The survival rate was significantly lower in the Mod group than in the Con group, but was similar in the Mod and ModGly groups. (b) The colonic weight-to-length ratio was greater in the Mod group than in the Con group and was the same in the Mod and ModGly groups. The data are presented as means ± SD; ^∗^*P* < 0.05, ^∗∗^*P* < 0.01, ^∗∗∗^*P* < 0.001.

We evaluated the effect of GLY on AA-induced colitis in mice by comparing the survival rate and the ratio of colonic weight to length. The survival rate in the Mod group was significantly lower than that in the CON group (Figure 1(a)), and the colonic weight/length ratio was increased (*P* < 0.05) (Figure 1(b)). However, Gly did not affect the survival rate or the colonic weight-to-length ratio in the ModGly group (Figures 1(a)–1(b)).

### 3.2. Serum Amino Acid Profile

**Figure 2:**
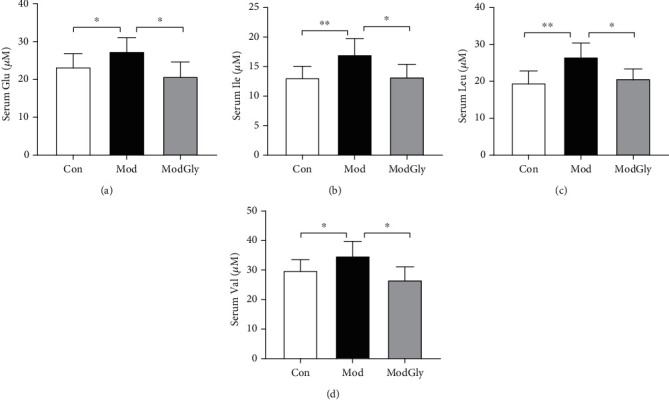
Serum amino acid profiles in the serum. The serum concentrations of Glu, glutamic acid (a), Ile, isoleucine (b), Leu, leucine (c), and Val, valine (d) were significantly higher (*P* < 0.05) in the Mod group than in the Con group and significantly lower (*P* < 0.05) in the Mod group than in the ModGly group. The data are presented as means ± SD; ^∗^*P* < 0.05, ^∗∗^*P* < 0.01, ^∗∗∗^*P* < 0.001.

The results showed that the serum leucine (Leu), isoleucine (Ile), valine (Val), and glutamic acid (Glu) concentrations were significantly higher (*P* < 0.05) in the Mod group than in the CON group (Figures 2(a)–2(d)), whereas the concentrations of other amino acids were largely unchanged (data not shown). Notably, dietary Gly reduced the serum concentrations of Leu, Ile, Val, and Glu in the Mod group.

### 3.3. Colonic Expression of IBD-Associated Cytokines

**Figure 3:**
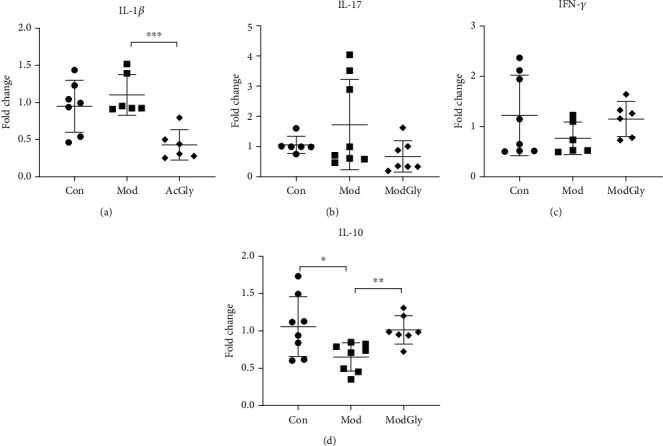
Colonic expression of IBD-associated cytokines. Real time-polymerase chain reaction analysis was used to measure the colonic expression of IL-1*β* (a), IL-17 (b), IFN-*γ* (c), and IL-10 (d) genes. AA administration reduced IL-10 expression, whereas Gly inhibited IL-1*β* expression and promoted IL-10 expression. The data are presented as means ± SD; ^∗^*P* < 0.05, ^∗∗^*P* < 0.01, ^∗∗∗^*P* < 0.001.

We analyzed the gene expression of colonic proinflammatory cytokines (such as IL-1*β*, IFN-*γ*, and IL-17) and inhibitory cytokine (IL-10). Compared with the CON group, the MOD group had significantly reduced expression of IL-10 (*P* < 0.05) (Figure 3(d)) but had an unaltered expression of proinflammatory cytokines (Figures 3(a)–(c)). In contrast, the ModGly group had significantly increased IL-10 expression (Figure 3(d)) and reduced IL-1*β* expression (Figure 3(c)).

### 3.4. Intestinal Microbiota

**Figure 4:**
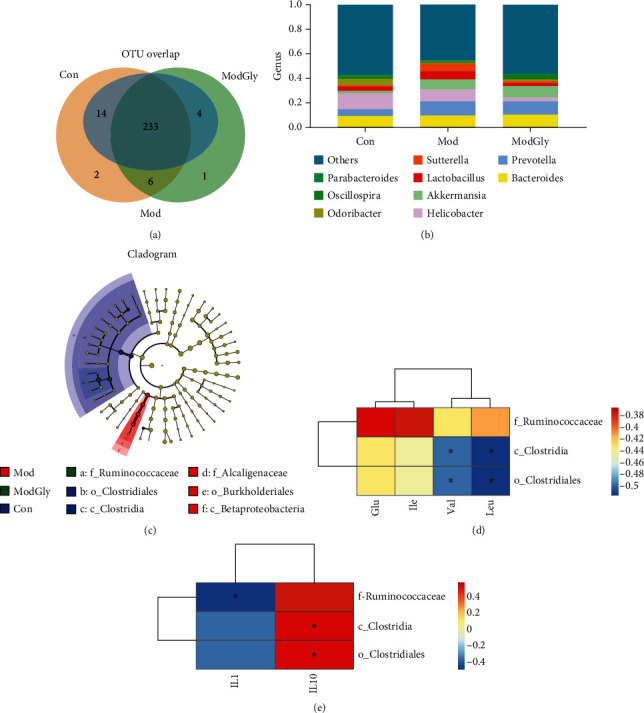
Intestinal microbiota. (a) Two hundred thirty-three OTUs were common to all three groups. (b) Relative contribution of genera in each group. (c) Prominent populations in each: Clostridia and Clostridiales in the Con group, Alcaligenaceae, Burkholderiales, and Betaproteobacteria in the Mod group, and Ruminococcaceae in the ModGly group. (d) The correlation analysis showed that serum concentrations of valine and leucine were negatively correlated with the abundance of Clostridia and Clostridiales (e); IL-10 expression was positively correlated with the abundance of Clostridia, Clostridiales, and IL-1 expression was negatively correlated with the abundance of Ruminococcaceae.

16sRNA was analyzed by Illumina high-throughput sequencing to examine the effect of the experimental conditions on the gut microbiota of mice. A total of 260 OTUs were found overall, and 233 of these OTUs were present in all three groups (Figure 4(a)). Twenty-six taxa were detected at the genus level, such as *Bacteroides*, *Prevotella*, *Helicobacter*, *Akkermansia*, *Lactobacillus*, and *Sutterella* (Figure 4(b)). Clostridia, Clostridiales, and Ruminococcaceae were relatively more abundant in both the Con and ModGly groups than in the Mod group (Table 2). The prominent populations in each group were also determined based on linear discriminant analysis coupled with effect-size measurements, which revealed that Clostridia and Clostridiales were dominant in the Con group, Alcaligenaceae, Burkholderiales, and Betaproteobacteria were dominant in the Mod group, and Ruminococcaceae were dominant in the ModGly group (Figure 4(c)). A correlation analysis showed that the serum concentrations of Val and Leu were negatively correlated with the abundance of Clostridia and Clostridiales (Figure 4(d)). Moreover, colonic IL-10 expression was positively correlated with the abundance of Clostridia, Clostridiales, and IL-1 expression was negatively correlated with the abundance of Ruminococcaceae (Figure 4(e)).

## 4. Discussion

Previous studies have shown that body weight, survival rates, and the ratio of colon weight to colon length decreased in the AA-induced IBD mouse model [17, 21]. It has also been shown that the expression of proinflammatory cytokines (i.e., IL-1*β*, IFN-*γ*, and IL-17) is significantly increased in the AA-induced IBD model. Intriguingly, it has been found that dietary supplementation Gly partially restores the above indicators in Wistar rat models of TNBS-or DSS-induced IBD [17]. However, we found that dietary Gly did not have a significant effect on survival rate and colonic weight-to-length ratio in the mouse model with AA-induced IBD. This different result may be attributable to the mouse model and/or the dosage of Gly we used.

Amino acids are necessary for intestinal growth and for the maintenance of mucosal integrity and barrier function [22–24]. Dietary supplementation with amino acids, such as threonine, serine, proline, and cysteine, improves mucin production, protects intestinal epithelial cells, and enhances the health of the intestinal microbiome [25–27]. Some amino acids have been suggested to be suitable as biomarkers for diagnosis and treatment in IBD, such as branched-chain amino acids (BCAAs; i.e., Leu, Ile, and Val) and tryptophan [28]. Concentrations of BCAAs and Glu have also been found to be significantly increased in a model of IBD [7], and these increases could be reversed by dietary Gly. Studies have also reported that BCAAs enhance the intestinal immune system by improving the morphological integrity of the intestinal tract and increasing the production of immunoglobulin. For example, Leu enhances cell proliferation and amino-acid transporter expression by activating the mTOR pathway [29]. However, a high concentration of BCAAs may activate the mTOR and NF-*κ*B pathways and thereby increase oxidative stress and inflammation. Other non-BCAAs have shown beneficial effects: for example, arginine reduces mucosal permeability, inhibits inflammation, and increases iNOS activity in mice with DSS-induced colitis [15]. Moreover, Gly inhibits cytokine secretion in monocytes, macrophages, and neutrophils and regulates T cell activities [16].

Current evidence suggests that immune disorders contribute to the IBD process via cytokine production in bowel lesions. Many proinflammatory cytokines, such as TNF-*α* and IL-1/6/22/23, and anti-inflammatory cytokines (such as IL-10) have been studied to determine their roles in IBD [30]. For example, it was found that IL-1 is highly activated in IBD patients and that IL-1*β* activates CD4+ T cells [31]. It was also found that mice that the lack of IL-10 or IL-10R are sensitive to colitis [32], largely due to the promotion of IL-1 production.

In another study, it was found that dietary Gly supplementation could reverse the increase of colonic IL-1*β* and the induction of TNF-*α* gene expression triggered by neutrophil-produced chemokines and inflammatory proteins secreted by macrophages [17]. Similarly, we found in this study that dietary Gly supplementation inhibited IL-1 expression and promoted IL-10 expression (as shown by increased IL-10 mRNA abundance), suggesting the potential of Gly for IBD treatment.

The gut microbiome has been linked to many diseases, such as IBD, obesity, diabetes, and autism [33]. Some studies have reported dramatic changes in the composition of the intestinal microbial community in mice with IBD, which is in keeping with our results. Dietary supplements, such as amino acids, may regulate physiological activity via their effects on gut microbes [34, 35]. In addition, a low-Gly diet is associated with changes in gut flora [36]. In this study, we found that Gly changed the composition of the microbiota, and this has associations with serum concentrations of amino acids and colonic gene expression of IL-1 and IL-10 in IBD models.

Interestingly, *Clostridium butyricum*, a member of Clostridia and Clostridiales, has been reported to promote the growth of gut IL-10-producing innate immune cells and alleviate colitis in a mouse model by activating Treg and inhibiting activated macrophages [37]. The findings indicate that dietary Gly regulates colonic cytokine expressions, especially that of IL-10, possibly via effects on gut bacteria. However, further studies are needed to investigate this possible mechanism.

In conclusion, our data show that dietary Gly modulated serum amino acid concentrations and gut cytokine expression in AA-induced colitis mice and that this may have occurred via the regulation of the intestinal microbiota. These findings enhance our understanding of the roles of Gly in the metabolism of serum amino acids, intestinal immunity, and its effects on the intestinal microbiota of mice.

## Figures and Tables

**Table 1:** Primers used in this study.

Gene	ID	Nucleotide sequence of primers (5′–3′)	Product length
IL-1*β*	NM_008361.3	ATGAAAGACGGCACACCCAC GCTTGTGCTCTGCTTGTGAG	175
IL-17	NM_010552.3	TACCTCAACCGTTCCACGTC TTTCCCTCCGCATTGACAC	119
IL-10	NM_010548.2	GCCACATGCTCCTAGAGCTG CAGCTGGTCCTTTGTTTGAAA	71
IFN-*γ*	NM_008337.4	ATGAACGCTACACACTGCATCTTGGCTT CCTCAAACTTGGCAATACTCATGAATGC	361
Actin	NM_007393.5	GGACTCCTATGTGGGTGACGAGG GGGAGAGCATAGCCCTCGTAGAT	366

**Table 2:** Taxa with significantly changed relative abundances at the genus level.

	Con	Mod	Gly
Clostridia	0.21380^∗^	0.07538	0.1699^#^
Ruminococcaceae	0.03527^∗^	0.01695	0.06516^#^
Clostridiales	0.2138^∗^	0.07538	0.2130^#^

The relative abundances of Clostridia, Clostridiales, and Ruminococcaceae were significantly higher in both the Con and ModGly groups than in the Mod group. The data are presented as means ± SD; ^∗^*P* < 0.05, Con vs. Mod; ^#^*P* < 0.05, ModGly vs. Mod.

## Data Availability

The data used to support the findings of this study are available from the corresponding author upon request.
